# SIRT3 Deficiency Induces Endothelial Insulin Resistance and Blunts Endothelial-Dependent Vasorelaxation in Mice and Human with Obesity

**DOI:** 10.1038/srep23366

**Published:** 2016-03-22

**Authors:** Lu Yang, Julei Zhang, Wenjuan Xing, Xing Zhang, Jie Xu, Haifeng Zhang, Li Chen, Xiaona Ning, Gang Ji, Jia Li, Qingchuan Zhao, Feng Gao

**Affiliations:** 1 Department of Aerospace Medicine, Fourth Military Medical University, 169 Changlexi Road, Xi’an 710032, China; 2 Department of Physiology, Fourth Military Medical University, 169 Changlexi Road, Xi’an 710032, China; 3 Department of Cardiology, Xijing Hospital, Fourth Military Medical University, 169 Changlexi Road, Xi’an 710032, China; 4 Department of Digestive Diseases, Xijing Hospital, Fourth Military Medical University, 169 Changlexi Road, Xi’an 710032, China

## Abstract

Recent evidence implicates the critical role of Sirtuin 3 (SIRT3) in the development of many metabolic diseases, but the contribution of SIRT3 to vascular homeostasis remains largely unknown. The aim of this study was to investigate the role of SIRT3 in endothelial insulin resistance and vascular dysfunction in obesity. We found an impaired insulin-induced mesenteric vasorelaxation and concomitant reduced vascular SIRT3 expression in morbid obese human subjects compared with the non-obese subjects. Downregulation of SIRT3 in cultured human endothelial cells increased mitochondrial reactive oxygen species (mtROS) and impaired insulin signaling as evidenced by decreased phosphorylation of Akt and endothelial nitric oxide synthase and subsequent reduced nitric oxide (NO) release. In addition, obese mice induced by 24-week high-fat diet (HFD) displayed an impaired endothelium-dependent vasorelaxation to both insulin and acetylcholine, which was further exacerbated by the gene deletion of *Sirt3*. Scavenging of mtROS not only restored insulin-stimulated NO production in SIRT3 knockdown cells, but also improved insulin-induced vasorelaxation in SIRT3 knockout mice fed with HFD. Taken together, our findings suggest that SIRT3 positively regulates endothelial insulin sensitivity and show that SIRT3 deficiency and resultant increased mtROS contribute to vascular dysfunction in obesity.

Obesity represents one of the main health problems in modern societies and has been related to insulin resistance and increased cardiovascular diseases^1^. Recent evidence from animal studies has shown that insulin resistance plays a common causal role in the development of endothelial dysfunction^2^,^3^,^4^, an early step in the pathogenesis of hypertension and atherosclerosis^5^. Insulin, in addition to its essential metabolic modulation, has important vascular actions including stimulating endothelial nitric oxide (NO) production, leading to vasodilation and increased tissue perfusion^6^. The integrity of endothelial cell insulin signaling plays a critical role in determining vessel endothelial function and NO bioavailability in response to conventional agonists^7^. Therapeutic interventions that improve endothelial function and/or insulin sensitivity attenuate metabolic and cardiovascular abnormalities in animal and clinical investigations^8^,^9^,^10^. However, the upstream molecules regulating endothelial insulin sensitivity have not been clarified.

Mitochondrial reactive oxygen species (mtROS) has recently been implicated as key regulators of vascular homeostatic functions in physiology and disease^11^. They contribute to endothelium-dependent vasodilation, whereas excessive mtROS generation is considered to cause deleterious vascular cell signaling and subsequent endothelial dysfunction^12^,^13^,^14^,^15^,^16^. Sirtuin 3 (SIRT3), a mitochondrial NAD^+^-dependent deacetylase that governs mitochondrial metabolism and ROS homeostasis, has been reported to be closel**y** involved in the regulation of mitochondrial function in metabolic syndrome, aging and pulmonary arterial hypertension^17^,^18^,^19^,^20^. SIRT3 activity is increased by nutrient distress such as fasting and caloric restriction, while mice lacking SIRT3 fed with a high-fat diet showed accelerated obesity and insulin resistance^21^,^22^. Recent studies reported that SIRT3 protects endothelial cells from high glucose-induced cytotoxicity and Ang II-induced endothelial dysfunction^23^,^24^. However, the role of SIRT3 in obesity associated endothelial dysfunction remains unknown. In this study, we found the impaired insulin-induced mesenteric vasorelaxation that is associated with reduced vascular SIRT3 expression in obese patients. We further identified SIRT3 as a critical player in obesity-induced vascular insulin resistance and resultant endothelial dysfunction.

## Results

### Vascular SIRT3 expression and insulin-induced vasodilation were subdued in morbid obese human subjects

**Figure 1:**
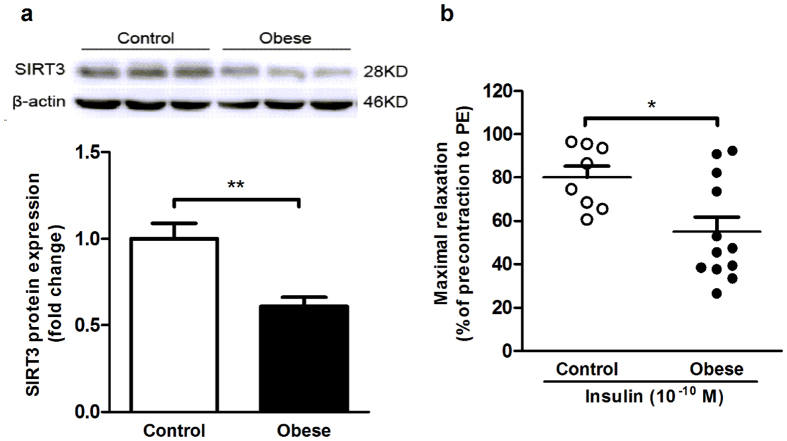
Vascular SIRT3 expression and insulin-induced vasodilation were subdued in morbid obese human subjects. Small arteries (200–500 μm) were isolated from visceral fat obtained from morbid obese or non-obese control subjects during surgery. (**a**) Western blot and quantitation of SIRT3 protein expression in vascular lysates from morbid obese and control subjects. (**b**) Relaxation response to insulin (10^−10^ M) was blunted in vessels from morbid obese subjects, which are indicated as percent relaxation of the precontraction to phenylephrine (PE) (10^−5 ^M). All values are presented as mean ± SEM. **P < 0.01 versus Control; ns indicates no significance. n = 8 to 12 per group (4 rings for each human patient).

We firstly observed SIRT3 expression in small mesenteric arteries from insulin-resistant morbid obese subjects and non-obese subjects with normal insulin sensitivity. As shown in Fig. 1a, vascular SIRT3 protein expression was significantly lower in morbid obese subjects compared with non-obese control subjects. More importantly, small mesenteric arteries from morbid obese subjects showed a significant reduction in the vasodilation response to insulin at a physiological concentration of 10^−10^ M compared with non-obese subjects (Fig. 1b). These results demonstrated that both SIRT3 expression and endothelial-dependent vasorelaxation to insulin were subdued in small arteries from morbid obese human subjects.

### Downregulation of SIRT3 was associated with reduced endothelial insulin sensitivity

**Figure 2:**
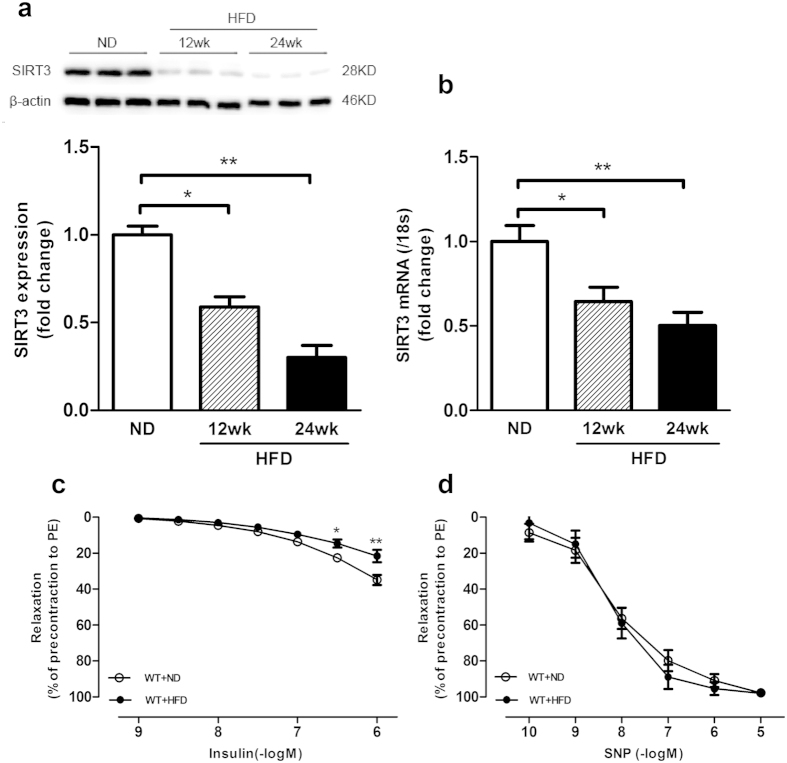
Vascular SIRT3 expression was downregulated in obese mice. (**a**,**b**) SIRT3 mRNA expression and protein level were quantified by real time PCR and Western blot in aortic lysates from mice fed with normal diet (ND) for 12 weeks or high-fat diet (HFD, 45% fat) for 12 and 24 weeks. (**c**,**d**) Vascular relaxation to insulin and nitric oxide donor sodium nitroprusside (SNP) in aorta from mice fed with ND or HFD for 24 weeks, which are expressed as percent relaxation of the precontraction to phenylephrine (PE) (10^−5^ M). Repeated-measures ANOVA were used to compare vascular dose-response curves to pharmacological probes. All values are presented as mean ± SEM. *P < 0.05, **P < 0.01 versus WT + HFD group. n = 10 to 12 mice per group (4 rings for each mouse).

We further examined SIRT3 expression in the thoracic aorta obtained from obese mice induced by 24 weeks of high-fat diet (HFD) feeding. Mice fed with 24 weeks of HFD displayed significantly higher body weight (34.89 ± 2.75 *vs*. 26.94 ± 2.11 g, n = 10–12, *P* < 0.05) and obvious obesity (epididymal fat: 1.74 ± 0.28 *vs*. 0.36 ± 0.08 g, n = 10–12, *P* < 0.05) compared with those fed with normal diet (ND). SIRT3 mRNA and protein expression significantly decreased in mice fed with 12 weeks of HFD and dropped below 50% after 24 weeks of HFD feeding compared with ND feeding (Fig. 2a,b). More importantly, there was a significantly reduced vasodilation response to insulin (Fig. 2c) in aorta from HFD-fed mice compared with ND-fed mice, while no difference was observed in the vasodilation response to SNP between the two groups (Fig. 2d)

**Figure 3:**
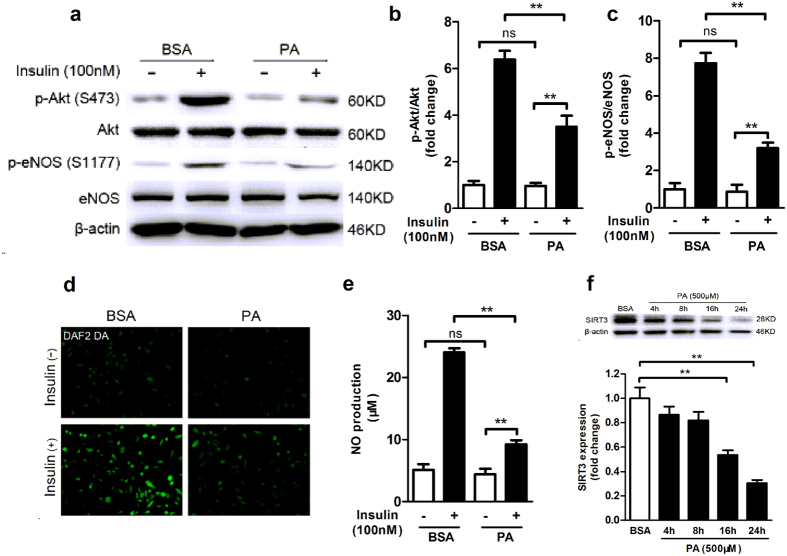
SIRT3 expression was downregulated in insulin-resistant endothelial cells. (**a**–**c**) Phosphorylated and total Akt and eNOS with or without insulin stimulation (100 nM, 20 min) in human umbilical vein endothelial cells (HUVECs) exposed to palmitate (PA, 500 μM) or vehicle for 24 h. (**d**) NO production as detected by DAF2 DA fluorescence in HUVECs (magnification, × 100). (**e**) NO level in cell media supernatant was detected by NO detection kit based on Griess method. (**f**) Western blot and quantitation of SIRT3 protein expression at different time points (4, 8, 16 and 24 h) of palmitate-treatment. All values are presented as mean ± SEM. *P < 0.05, **P < 0.01; ns indicates no significance. n = 4 independent experiments.

To investigate the potential correlation between SIRT3 downregulation and decreased endothelial-dependent response to insulin in vessels, human umbilical vein endothelial cells (HUVECs) were exposed to 500 μM palmitate for 24 h to mimic the cellular injury induced by HFD feeding. Western blot revealed that palmitate treatment had no effects on the basal phosphorylation or total protein levels of Akt and endothelial nitric oxide synthase (eNOS) in endothelial cells (Fig. 3a–c). However, under insulin stimulation, phosphorylation of Akt (Ser 473) and eNOS (Ser 1177) decreased by about 50% and 60% respectively, in palmitate-treated cells compared with vehicle-treated cells (Fig. 3a–c). As a result, insulin-stimulated NO production as detected by DAF2 DA (diaminofluorescein-2 diacetate) fluorescence significantly decreased in palmitate-treated cells (Fig. 3d), which was further confirmed by reduced NO level in the media supernatant of endothelial cells by using Griess method (Fig. 3e). We also investigated the change of SIRT3 expression in the endothelial cells at serial time points of palmitate-treatment (4, 8, 16 and 24 h). As shown in Fig. 3f, at 4 and 8 h of palmitate-exposure, there was a moderate but not significant reduction in SIRT3 expression and it decreased significantly by about 25% and 50% at 16 and 24 h of palmitate-exposure respectively, compared with vehicle-treated cells. Taken together, these results indicated that SIRT3 downregulation might be correlated with reduced insulin sensitivity in endothelial cells.

### SIRT3 is a positive regulator of endothelial insulin sensitivity

**Figure 4:**
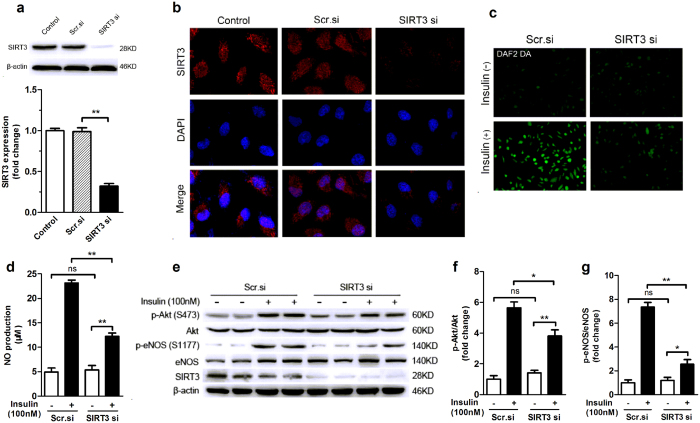
SIRT3 knockdown reduced insulin response in endothelial cells. (**a**,**b**) Representative Western blots and immunofluorescence staining of SIRT3 (red) in human umbilical vein endothelial cells (HUVECs) treated with scrambled small interfering RNA (Scr.si) or SIRT3 small interfering RNA (SIRT3 si). Nuclei were labeled with DAPI (blue) (magnification, ×400). (**c**) NO production as detected by DAF2 DA fluorescence (magnification, ×100) in HUVECs. (**d**) NO level in cell media supernatant was detected by NO detection kit based on Griess method. (**e–g**) Representative Western blot and densitometric quantification of Akt and eNOS phosphorylation with or without insulin stimulation (100 nM, 20 min) in HUVECs. All values are presented as mean ± SEM. *P < 0.05, **P < 0.01; ns indicates no significance. n = 4 independent experiments.

To elucidate the role of SIRT3 in regulating endothelial insulin sensitivity, we downregulated SIRT3 in HUVECs by using selective SIRT3 siRNA. This approach significantly reduced SIRT3 protein expression by about 80%, while the scrambled siRNA did not exert any effects (Fig. 4a). siRNA-mediated knockdown of SIRT3 was further confirmed by immunofluorescence analysis showing reduced SIRT3 staining in endothelial cells (Fig. 4b). Of interest, SIRT3 knockdown resulted in a significant decrease in insulin-stimulated Akt and eNOS phosphorylation in endothelial cells (Fig. 4e). Accordingly, a significantly reduced NO production was observed in SIRT3-knockdown cells, as detected by DAF2 DA fluorescence and Griess method, respectively (Fig. 4c,d). There were no significant differences in the total protein level of Akt and eNOS between SIRT3-knockdown and control cells (Fig. 4e–g). These data suggested that down-regulation of SIRT3 reduced insulin sensitivity in endothelial cells.

**Figure 5:**
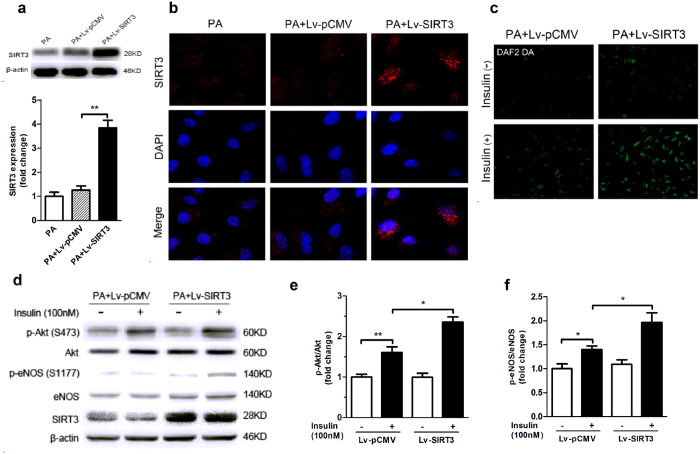
SIRT3 overexpression alleviated endothelial insulin resistance induced by palmitate treatment. (**a**,**b**) HUVECs were infected with lentivirus-SIRT3 (Lv-SIRT3) or negative control lentivirus-pCMV (Lv-pCMV). After 48 h of infection, the cells were exposed to palmitate (500 nM, PA) for 24 h and SIRT3 expression was analyzed by Western blot and immunofluorescence (magnification, × 400). (**c**) NO production was detected by DAF2 DA fluorescence (magnification, × 100). (**d–f**) Representative western blot and densitometric quantification of Akt and eNOS phosphorylation with or without insulin stimulation (100 nM, 20 min) in HUVECs which were infected with Lv-SIRT3 or negative control Lv-pCMV. All values are presented as mean ± SEM. *P < 0.05, **P < 0.01; ns indicates no significance. n = 4 independent experiments.

To further investigate whether SIRT3 overexpression exerts protective effects against palmitate-induced endothelial insulin resistance, HUVECs were infected with lentiviral (Lv)-SIRT3 before palmitate exposure. Endothelial cells infected with Lv-SIRT3 showed a significant increase in SIRT3 expression compared with Lv-pCMV-infected cells (Fig. 5a), which was confirmed by immunofluorescence analysis of SIRT3-specific staining (Fig. 5b). As shown in Fig. 5d–f, palmitate caused a significant inhibition on endothelial insulin response as evidenced by reduced Akt and eNOS phosphorylation and a consequent reduction in insulin-stimulated NO production (Fig. 5c). Overexpression of SIRT3 by Lv-SIRT3 infection abolished the palmitate-induced inhibition on both insulin signaling and insulin-stimulated NO production, suggesting that SIRT3 overexpression improves insulin resistance induced by palmitate in endothelial cells. The effects of SIRT3 on insulin signaling and NO production were further confirmed by the data from cultured human aortic endothelial cells (HAECs) (see Supplementary Fig. S1). Collectively, these results from both loss-and gain-of-function of SIRT3 indicated its positive role in regulating endothelial insulin sensitivity.

### SIRT3 deficiency exacerbated endothelial dysfunction in diet-induced obese mice

**Figure 6:**
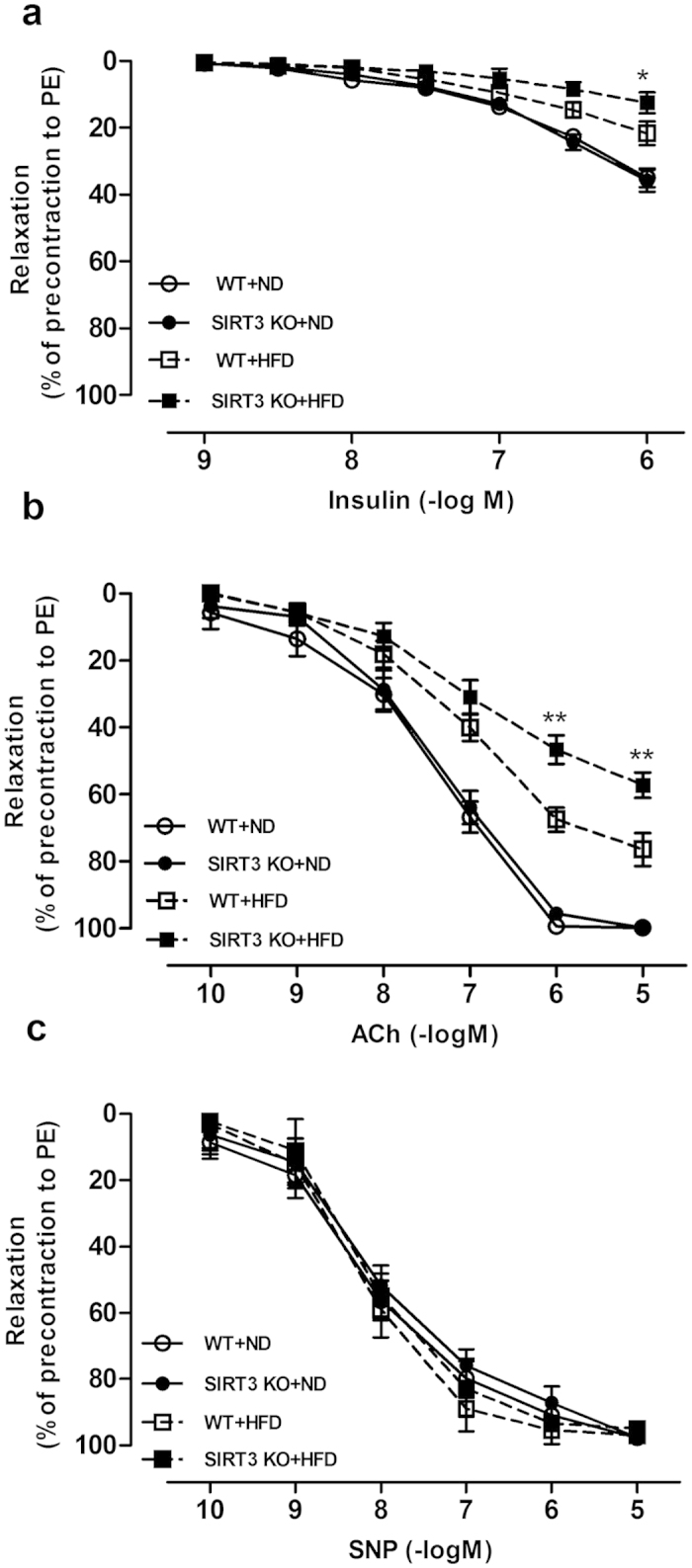
SIRT3 deficiency exacerbated endothelial dysfunction in diet-induced obese mice. Male SIRT3KO mice and wild type (WT) littermates aged 8 weeks were randomized into two groups and maintained on a standard normal diet (ND) or a high-fat diet (HFD, 45% fat) for 24 weeks before aorta were excised for analysis. (**a–c**) Vascular relaxation to insulin, acetylcholine (ACh) and nitric oxide donor sodium nitroprusside (SNP), which are expressed as percent relaxation of the precontraction to phenylephrine (PE) (10^−5^ M). Repeated-measures ANOVA was used to compare vascular dose-response curves to pharmacological probes. All values are presented as mean ± SEM. *P < 0.05, **P < 0.01 versus WT + HFD group. n = 10 to 12 mice per group (4 rings for each mouse).

To investigate the effect of *Sirt3* gene deletion on endothelial function *in vivo*, the vasoactive response was determined in the aorta of SIRT3 knockout (KO) and wide type (WT) mice fed with ND or HFD. There was no significant difference in vascular relaxation to insulin or acetylcholine (ACh) between SIRT3KO and WT mice on ND (Fig. 6a,b). However, obese mice induced by 24-week-HFD feeding displayed a significantly impaired vascular relaxation to both insulin and ACh (Fig. 6a,b), while SIRT3 deficiency exacerbated this impairment of vascular response to both insulin and ACh (Fig. 6a,b) in the HFD-induced obese mice. Endothelium-independent relaxation to SNP was similar among all groups (Fig. 6c). Metabolic characteristics and inflammatory cytokines of SIRT3KO and WT mice fed with ND or HFD for 24 weeks were recorded and provided in Supplementary Table. S1.

### Mitochondrial ROS was involved in the regulation of SIRT3 on endothelial insulin sensitivity

**Figure 7:**
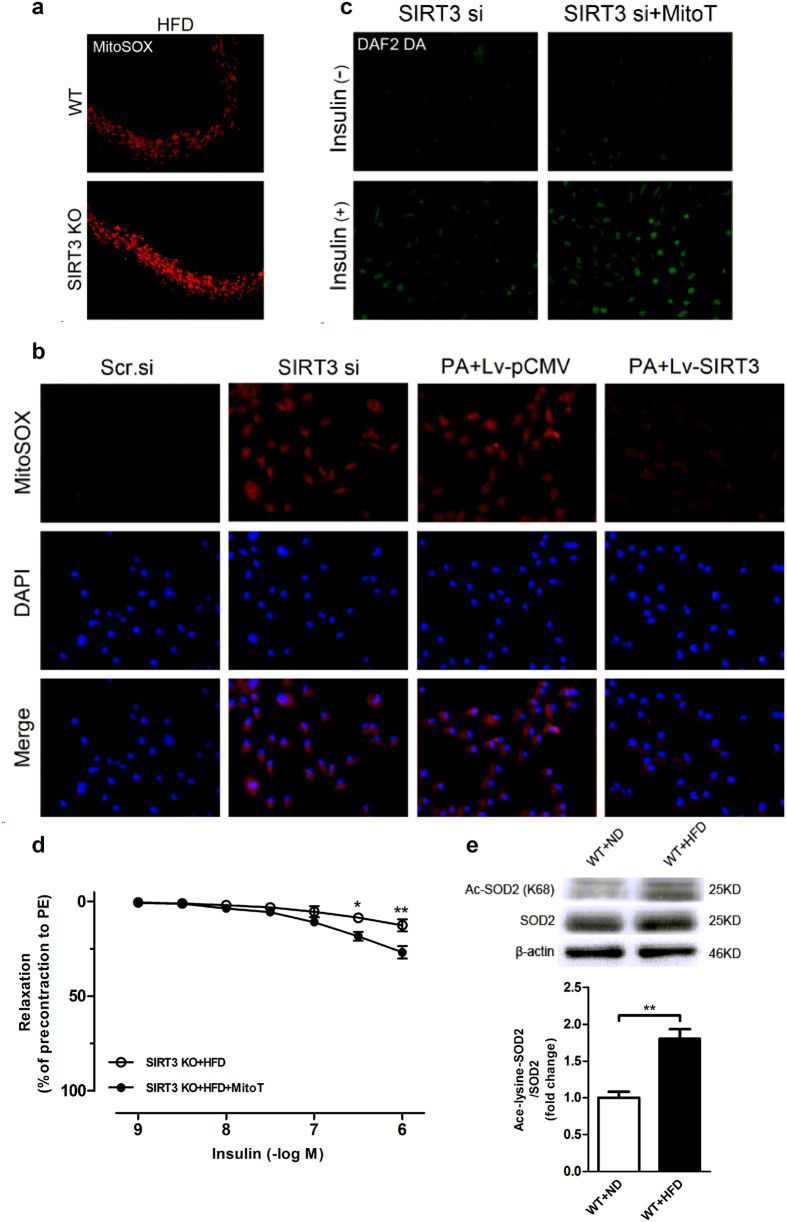
Mitochondrial ROS was involved in the regulation of SIRT3 on endothelial insulin sensitivity. (**a**) *In situ* mitochondrial O_2_^•−^ generation as detected by MitoSOX within the aortic rings from wide type (WT) and SIRT3 knockout (SIRT3KO) mice (magnification, ×100). (**b**) Human umbilical vein endothelial cells (HUVECs) were transfected with SIRT3 siRNA (SIRT3 si) for 48 h or infected with lentivirus-SIRT3 (Lv-SIRT3) for 72 h. The scrambled siRNA (Scr.si) and lentivirus-pCMV (Lv-pCMV) served as the negative control, respectively. Mitochondrial O_2_^•−^ generation was detected by MitoSOX staining (5 μM at 37 °C, 15 min) (magnification, ×100). (**c**) NO production was detected by DAF2 DA in SIRT3-knockdown HUVECs with or without MitoTEMPO treatment (MitoT, 100 μM) (magnification, ×100). (**d**) Incubation with MitoTEMPO (100 μM, 30 min) ameliorated HFD-induced impairment of vasorelaxation to insulin in SIRT3KO mice (n = 8). (**e**) The expression and acetylation of manganese superoxide dismutase (SOD2) were assessed by Western blot in vessel lysates from mice fed with ND or HFD for 24 weeks. Repeated-measures ANOVA was used to compare vascular dose-response curves to pharmacological probes. All values are presented as mean ± SEM. *P < 0.05, **P < 0.01 versus SIRT3KO + HFD + MitoT group. n = 4 to 6 mice per group (4 rings for each mouse) for A and E; n = 4 independent experiments for b and c.

To investigate the impact of *Sirt3* deletion on mtROS production, a mitochondrial specific oxidation-sensitive dye MitoSOX, was used to detect mitochondrial O_2_^•−^ generation^25^. As shown in Fig. 7a, a significant increase of mitochondrial O_2_^•−^ as detected by MitoSOX fluorescence was observed in the aorta from SIRT3KO mice fed with HFD compared with WT fed with HFD. siRNA-mediated knockdown of SIRT3 resulted in a significant elevation in mitochondrial O_2_^•−^ generation while overexpression of SIRT3 by Lv-SIRT3 infection significantly inhibited mitochondrial O_2_^•−^ increase induced by palmitate in endothelial cells (Fig. 7b). As described in Fig. 7c, SIRT3 knockdown led to a reduction in insulin-stimulated NO release. Interestingly, elimination of mtROS with MitoTEMPO (MitoT, a mitochondria-targeted superoxide dismutase) almost restored insulin-stimulated NO release in SIRT3 knockdown cells (Fig. 7c). In addition, pretreatment with MitoTEMPO significantly alleviated the impairment of endothelium-dependent relaxation to insulin in the aorta from SIRT3KO mice on HFD (Fig. 7d).

Manganese superoxide dismutase (SOD2) is the primary mitochondrial superoxide scavenging enzyme and it was uniformly shown that SOD2 was directly deacetylated by SIRT3 in *in vitro* and *in vivo* model systems^26^. As shown in Fig. 7e, acetylation (ac)-SOD2 (K68) was significantly increased while SOD2 expression was preserved in vessel lysates from mice fed with HFD compared with those from mice fed with ND. Collectively, all these data indicated that mtROS is involved in the regulation of SIRT3 on endothelial insulin sensitivity and SIRT3 protects against HFD-induced endothelial dysfunction by inhibiting mtROS increase.

## Discussion

The present study demonstrates for the first time that SIRT3 is a positive regulator of endothelial insulin sensitivity. SIRT3 deficiency and resultant mtROS overproduction contribute to endothelial dysfunction in obesity. Several lines of evidence support our conclusions. First, in obese patients there was an association between reduced SIRT3 expression and impaired insulin-induced vasorelaxation. Second, SIRT3 knockdown and the resultant mtROS increase led to endothelial impaired insulin signaling and reduced NO production, while overexpression of SIRT3 or elimination of mtROS improved insulin sensitivity in cultured endothelial cells. Third, obese mice induced by HFD exhibited an impaired vascular relaxation to both insulin and ACh, which was further exacerbated in mice with a genetic deletion of *Sirt3*. Scavenging of excess mtROS rescued HFD-induced endothelial dysfunction in SIRT3KO mice.

SIRT3 deficiency has been implicated in the development of metabolic syndrome, pulmonary arterial hypertension and human aging^19^,^22^,^27^. Recently, Winnik S *et al*. has reported that deletion of *Sirt3* accelerates weight gain and impairs rapid metabolic adaptation in low-density lipoprotein receptor knockout mice but does not further aggravate atherosclerosis^28^. In the present study, acute knockdown of SIRT3 by siRNA reduced endothelial response to insulin although no significant change of insulin receptor and PI3-kinase was detected in aorta from SIRT3KO mice compared with the WT mice. SIRT3KO mice showed no defects in vasorelaxation to insulin or ACh, which might be due to a developmental or compensatory difference caused by the lack of SIRT3 from birth. We also detected the expression of SIRT4 and SIRT5 in aorta from SIRT3KO mice and found no significant change compared with the WT mice, which implied no compensatory effect existed from other mitochondrial Sirtuins (see Supplementary Fig. S2). However, SIRT3KO mice during HFD-feeding exhibited aggravated impairment of endothelial-dependent vascular response to both insulin and ACh compared with their wild type counterparts. In contrast, overexpression of SIRT3 ameoliorated palmitate-induced endothelial insulin resistance. Moreover, we observed that insulin-induced mesenteric vasorelaxation was impaired and vascular SIRT3 expression was concurrently reduced in morbid obese patients. However, we cannot rule out the possibility that diabetes might contribute to endothelial dysfunction considering that the subjects with obesity also had higher fasting blood glucose and insulin levels compared with those in non-obese controls (Table 1). Further studies are ongoing to determine whether SIRT3 deficiency is also present in non-insulin resistant morbid obese patient. Taken together, these results suggest that SIRT3 is a positive regulator in endothelial insulin sensitivity and a protector against HFD-induced endothelial dysfunction.

Accumulating evidence indicates that oxidative stress plays a major role in the initiation and progression of endothelial dysfunction associated with diseases such as hyperlipidemia, diabetes mellitus, and hypertension^29^. Various sources of oxidative stress within the vascular endothelial cell include xanthine oxidase, NADPH (Nicotinamide adenine dinucleotide phosphate) oxidase, uncoupled eNOS, and the mitochondrial electron transport chain (mETC)^11^. Although increased NADPH oxidase expression and activity was found in human atherosclerotic vessels and diabetic vessels^29^, we found that *Sirt3* gene deletion had no significant effects on the vascular expression of NOX4 (NADPH oxidase 4) and p47phox (see Supplementary Fig. S3). A recent study has shown that altered mitochondrial dynamics in diabetes mellitus with increased production of ROS contributed to endothelial dysfunction in diabetes mellitus^15^. Our data showed that there was an increase of mitochondrial O_2_^•−^ in aortic rings from SIRT3KO mice fed with HFD, whereas a mitochondrial O_2_^•−^ specific scavenger MitoTEMPO significantly improved the endothelial-dependent vasorelaxation in these mice. In cultured endothelial cells, scavenging mitochondrial ROS significantly abolished the inhibition of NO production induced by SIRT3 knockdown. These results are consistent with the previous reports and provide further evidence that prevention of mitochondrial oxidative damage is a therapeutic strategy in endothelial dysfunction. Another possible mechanism for endothelial dysfunction is endothelial NO synthase (eNOS) uncoupling, whereby eNOS generates O_2_^•−^ rather than NO because of deficient eNOS cofactor tetrahydrobiopterin (BH4)^30^. Further studies are needed to investigate whether eNOS uncoupling is involved in this pathological process.

In conclusion, Our results showed that SIRT3 deficiency and resultant mtROS overproduction lead to endothelial insulin resistance and contribute to endothelial dysfunction in obesity. Overexpression of SIRT3 improves endothelial insulin sensitivity and attenuates endothelial dysfunction induced by nutrient excess through inhibiting mitochondrial oxidative stress. These findings reveal a novel role for SIRT3 in regulating endothelial insulin sensitivity and provide further compelling support for SIRT3 as a potential therapeutic target for pharmacological interventions aimed at the prevention and treatment of cardiovascular diseases related to nutrient excess.

## Methods

### Subjects

All procedures involving human tissue samples were approved by the Human Research Ethics Committee of Fourth Military Medical University. This study conformed to the principles outlined in the Declaration of Helsinki. Informed consent was obtained from all subjects. Small arteries (200–500 μm, approximately 2 mm length) were isolated from visceral fat obtained from insulin-resistant morbid obese and non-obese human subjects during surgery. Insulin resistance was estimated by calculating the score for Homeostasis Model Assessment of Insulin Resistance (HOMA-IR)^31^. Twelve morbid obese subjects underwent bariatric surgery in Xijing Hospital, with a body mass index (BMI) ≥ 30 kg/m^2^, HOMA-IR ≥ 3.8. Subjects with history or clinical evidence of cardiovascular diseases (congestive heart failure, ischemic heart disease and hypertension) were excluded, but not those with type 2 diabetes or dyslipidemia. Control samples were obtained from the eight subjects (BMI < 30 kg/m^2^, HOMA-IR < 3.8) who underwent abdominal surgery (hiatus hernia repair, achalasia or cholecystectomies). Exclusion criteria for this control group were: diabetes, hypertension, history or clinical evidence of cardiovascular disease and any other conditions that might interfere with the progress of the study. The waist, hip circumference, systolic blood pressure (SBP) and diastolic blood pressure (DBP) were recorded when patients were hospitalized. The detailed patients’ characteristics are listed in Table 1.

### Animals

SIRT3 knock-out (SIRT3KO) and wild-type (WT) 129Sv mice were obtained from Jackson Laboratory (Bar Harbor, Maine), and created by Lombard *et al*.^32^. They were housed in temperature-controlled cages (20 °C to 22 °C, fed ad libitum, and maintained on a 12 h light /12 h dark cycle). At the age of 8 week, mice were randomized into two groups and maintained on a standard normal diet (ND) or a high-fat diet (HFD) (45% fat) for 24 weeks. The normal diet contained 3.85 kcal in each gram of food, of which there were 20% kcal from protein, 70% kcal from carbohydrates and 10% kcal from fat. The high-fat diet contained 4.73 kcal in each gram of food, of which there were 20% kcal from protein, 35% kcal from carbohydrates and 45% kcal from fat (Medicience Ltd., China). The mice were anaesthetized with the intraperitoneal administration of pentobarbital sodium (60 mg/kg) followed by vascular function test and biochemistry analysis. All procedures involving animals were performed according to the National Institutes of Health Guidelines on the Use of Laboratory Animals (NIH publication No. 85–23, revised 1996), and were approved by the Fourth Military Medical University Committee on Animal Care.

### Determination of blood variables in human

Serum concentrations of hsCRP (high-sensitivity C-reactive protein), SAA (Serum amyloid A) and MDA (Malonaldehyde) were determined by the corresponding assay kits (Nanjing Jiancheng Bioengineering Institute, China) according to the manufacturer’s instructions. Serum contents of IL-6 (Interleukin 6) and TNF-α (tumor necrosis factor alpha) were measured using human enzyme-linked immunosorbent assay kits (Beijing North Institute of Biological Technology, China) according to the manufacturer’s instructions.

### Vascular reactivity

Mice were sacrificed and the descending aorta was carefully excised and placed in ice-cold physiological saline solution (PSS) as described previously^33^. The contractile force was recorded using a PowerLab Chart v 7.2.1 program (model 610 M, Danish Myo Technology, Denmark). After a 40 min equilibration period, one dose of physiological salt solution containing 60 mM KCl (KPSS) was administered to verify vascular smooth muscle viability. Aortic rings were precontracted with phenylephrine (PE, 10^−5^ M, Sigma, USA). Endothelium-dependent vasorelaxation evoked by acetylcholine (ACh, 10^−10^ to 10^−5^ M, Sigma, USA) and insulin (10^−9^ to 10^−6^ M, Sigma, USA) and endothelium-independent vasorelaxation evoked by cumulative sodium nitroprusside (SNP, 10^−10^ to 10^−5^ M, Sigma, USA) were expressed as percent contraction determined by the percentage of inhibition to the precontracted tension. For scavenging of mtROS, MitoTEMPO (2-(2,2,6,6-tetramethyl-piperidin-1-oxyl-4-ylamino)-2-oxoethyl)-TPP, MitoT, 100 μM), a mitochondria-targeted superoxide dismutase mimetic, was added to the organ chamber 30 min before the assessment of vascular function.

### Western blot

Proteins were separated on a 10% SDS-PAGE gels, transferred to PVDF membrane (polyvinylidenedifluoride, Millipore, USA), and incubated overnight at 4 °C with antibodies directed against SIRT3, SOD2, Akt, phospho(p)-Akt (Ser 473) (1:1,000, Cell Signaling Technology, USA), acetylated(Ac)-SOD2 (Lys68) antibody (1:1,000, Abcam, USA), p-eNOS (Ser 1177), eNOS (1:1,000, BD Biosciences, USA) or β-actin (1:1,000, Santa Cruz, USA). After washing blots to remove excessive primary antibody binding, blots were incubated for 1 h with horseradish peroxidase (HRP)-conjugated secondary antibody (1:5,000, Boster, China) at room temperature. The blots were developed with an enhanced chemiluminescence detection kit (Roche, USA). The immunoblot was visualized with ChemiDocXRS (Bio-Rad, Hercules, USA) and the blot densities were analyzed with Lab Image software.

### Real-time PCR

The real-time PCR experiment was performed according to the MIQE guidelines^34^. Total RNA was extracted from flash-frozen tissue using TRIzol (Invitrogen, USA) and cDNA was synthesized from 1 μg RNA via reverse transcription reagent kit (TaKaRa, China). Expression analysis of the reported genes was performed by real-time PCR by PCR detection kit (TaKaRa) and BioRad Real-Time PCR Detection Systems. *18s* served as an endogenous control. The *Sirt3* cDNA was amplified with a pair of primers (reverse 5′-CAACATGAAAAAGGGCTTGGG-3′ and forward 5′-ATCCCGGACTTCAGATCCCC-3′). The *18s* cDNA was amplified with a pair of primers (reverse 5′-GACCTTGCTCCTTATTGAAGC-3′ and forward 5′-GACCTGCCTTACGACTATGG-3′) (Sangon, China).

### Cell culture

Human umbilical vein endothelial cells (HUVECs, Cell Applications, USA) were cultured in endothelial growth medium-2 (EGM-2) containing 5% fetal bovine serum (FBS) and SingleQuot Kit supplements (Lonza, USA). The cells were cultured at 37 °C in a humidified atmosphere containing 5% CO_2_, with medium changes three times a week. HUVECs come from the same donor and experiments performed on HUVECs were carried out at the 3–7 passages. The cells were synchronized in serum free EGM-2 for 6 h before all of the following treatments. The cells at 70–80% confluence were exposed to palmitate (500 μM; Sigma, USA) or bovine serum albumin (BSA; MP Biomedicals, USA) for 24 h. For the insulin stimulation experiments, the cells were treated with or without 100 nM insulin for 20 min. For lentivirus construction and infection, the coding region of the *Sirt3* gene was amplified by real time PCR and inserted downstream of the pCMV promoter in the lentiviral vector (GenePharma, China) to produce lentivirus (Lv)-SIRT3. The titer of lentivirus was 10^9^ pfu/mL. The HUVECs cultured at 60–70% confluence were infected with Lv-SIRT3 or control Lv-pCMV at a multiplicity of infection (MOI) of 10 in EGM-2 supplemented with 5% FBS according to the protocols provided by the manufacturer.

### siRNA transfection

Three candidate small interfering RNA (siRNA) specifically targeting SIRT3 mRNA and their scrambled RNA were purchased from GenePharma (China) and transfected into HUVECs with Lipofectamine 2000 (Invitrogen, USA) according to the protocols provided by the manufacturer. The siRNA sequences are as follows: human SIRT3, 1. sense 5′- CCAGCAUGAAAUACAUUUATT -3′, anti-sense 5′- UAAAUGUAUUUCAUGCUGGTT -3′. 2. sense 5′- CCAGUGGCAUUCCAGACUUTT -3′, anti-sense 5′- AAGUCUGGAAUGCCACUGGTT -3′. Scrambled siRNA, sense 5′- UUCUCCGAACGUGUCACGUTT -3′, anti-sense 5′-ACGUGACACGUUCGGAGAATT -3′. The transfected cells were cultured for 48 h and used for further experiments.

### Immunofluorescence

To localize SIRT3 expression within cultured HUVECs, immunofluorescent staining was performed. Cells grown on cover-slips were washed with phosphate buffered saline (PBS) three times and fixed in 4% paraformaldehyde before permeabilization with 0.1% Triton X-100 for 5 min. After washing three times, cells were blocked with 10% sheep serum (Sigma, USA) at room temperature for 1 h. The cover-slips were washed and incubated overnight at 4 °C with anti-SIRT3 (1:50, Cell Signaling Technology, USA). The cells were washed three times with PBS and incubated Cy3-labeled antibodies (1:200, Boster, China) for 1 h at room temperature. Nuclei were stained with 4′,6-diamidino-2-phenylindole (DAPI, 1 μmol/L; Beyotime Institute of Biotechnology, China) for 15 min. Images were acquired using a confocal microscope (FV1000, Olympus, Japan).

### Detection of mitochondrial superoxide production

The mitochondria-targeted O_2_^•−^-sensitive fluorophore MitoSOX Red (Molecular Probes, USA) was used as described previously^35^. Cells were cultured in low-absorbance 6-well plates (Corning, USA) and were washed with PBS three times before incubated with 5 μM MitoSOX at 37 °C for 15 min. After washing three times with PBS, nuclei were stained with DAPI (1 μM) for 15 min. Cells were imaged using a confocal microscope (FV1000, Olympus, Japan) with excitation at 390–485 nm and emission settings at 590–675 nm for MitoSOX.

### Measurement of nitric oxide level

Intracellular NO was examined in HUVECs loaded with NO sensitive fluorescent dye diaminofluorescein-2 diacetate (DAF2 DA; 5 μM, Sigma, USA) at 37 °C for 15 min^36^. After being washed three times with PBS, cells were imaged by a Nikon Eclipse Ti-E inverted fluorescence microscope with excitation at 450–495 nm and emission at 495–570 nm.

Total nitric oxide production (NO×) in media supernatant of cultured cells was determined by measuring the concentration of nitrite, a stable metabolite of NO with a modified Griess reaction method (Beyotime Institute of Biotechnology, China)^37^.

### Statistical analysis

All values in the text and figures are presented as means ± SEM of n independent experiments. Data (except Western blot density) were subjected to t test (two groups) or ANOVA (three or more groups) followed by Bonferonni correction for post hoc t-test. Vascular dose-response curves to pharmacological probes were analyzed using repeated-measures ANOVA. Western blot densities were analyzed with the Kruskal-Wallis test followed by Dunn’s post test. All of the statistical tests were performed with the GraphPad Prism software version 5.0 (GraphPad Software, Inc., USA) and probabilities of < 0.05 were considered to be statistically significant.

## Additional Information

**How to cite this article**: Yang, L. *et al*. SIRT3 Deficiency Induces Endothelial Insulin Resistance and Blunts Endothelial-Dependent Vasorelaxation in Mice and Human with Obesity. *Sci. Rep.* **6**, 23366; doi: 10.1038/srep23366 (2016).

## Supplementary Material

Supplementary Information

## Figures and Tables

**Table 1:** Basal variables of human subjects.

Characteristic	Control	Obesity	P value
	**(N = 8)**	**(N = 12)**	
Age (years)	45.75 ± 1.83	38.33 ± 2.98	ns
Gender (M/F)	4/4	4/8	ns
Height (cm)	169.88 ± 8.13	168.42 ± 4.65	ns
Body weight (kg)	63.25 ± 13.25	108.42 ± 14.33	<0.01
BMI	21.61 ± 2.55	38.05 ± 3.3	<0.01
Waist (cm)	85.74 ± 2.14	113.14 ± 9.88	<0.01
Hip circumference (cm)	104.6 ± 4.85	116.14 ± 5.84	<0.01
SBP (mmHg)	127.2 ± 7.45	130.57 ± 14.12	ns
DBP (mmHg)	86.13 ± 4.64	85 ± 7.14	ns
Fasting blood glucose (mmol/L)	5.3 ± 1.6	8.57 ± 3.73	<0.05
Fasting insulin (mIU/L)	9.43 ± 5.64	26.39 ± 13	<0.05
HOMR-IR	2.25 ± 0.41	9.61 ± 6.14	<0.05
Inflammation and oxidative stress			
hsCRP (mg/l)	1.36 ± 0.62	4.7 ± 0.84	<0.01
SAA (mg/l)	3.93 ± 0.72	5.82 ± 1.95	ns
IL-6 (pg/ml)	1.36 ± 0.32	5.14 ± 0.69	<0.001
TNF-α (pg/ml)	4.4 ± 0.63	7.12 ± 0.94	<0.05
MDA (μmol/l)	1.25 ± 0.29	2.86 ± 0.53	<0.05

M, male; F, female; BMI, body mass index; SBP, systolic blood pressure; DBP, diastolic blood pressure; HOMR-IR, homeostasis model assessment of insulin resistance; hsCRP, high-sensitivity C-reactive protein; SAA, serum amyloid A; IL-6, interleukin 6; TNF-α, tumor necrosis factor alpha; MDA, malonaldehyde; ns indicates no significance.
