# Supplementary material for: SIRT3 Deficiency Induces Endothelial Insulin Resistance and Blunts Endothelial-Dependent Vasorelaxation in Mice and Human with Obesity
Source: Sci Rep. 2016 Mar 22;6:23366. doi: 10.1038/srep23366 (PMC4802313; doi:10.1038/srep23366)
Supplement: Supplementary Information [file srep23366-s1.doc]

**SIRT3 Deficiency Induces Endothelial Insulin Resistance and Blunts Endothelial-Dependent Vasorelaxation in Mice and Human with Obesity**

Lu Yang, Julei Zhang, Wenjuan Xing, Xing Zhang, Jie Xu, Haifeng Zhang, Li Chen, Xiaona Ning, Jia Li, Qingchuan Zhao, Feng Gao

**Supplementary materials**

**Determination of blood variables in mice**

Plasma cholesterol and triglyceride levels were determined by a biochemistry analyzer in Xijing hospital (Cobas Integra 400 Plus, Roche, Germany). Fasting blood glucose level was measured using a glucose meter (Life-Scan, USA). Serum insulin levels were measured using commercial radioimmunoassay kits (Beijing North Institute of Biological Technology, China). Serum contents of IL-6 and TNF-α were measured using mouse enzyme-linked immunosorbent assay kits (Merck-Millipore, Germany) according to the manufacturer’s instructions.

**Supplementary Table 1.** Plasma lipids and inflammatory cytokines of SIRT3KO and WT mice fed with normal diet or high-fat diet for 24 weeks.

|  | **WT** | | **SIRT3KO** | |
| --- | --- | --- | --- | --- |
|  | **ND** | **HFD** | **ND** | **HFD** |
| **Body weight (g)** | 26.9 ± 2.1 | 34.9 ± 2.8* | 25.3 ± 2.3 | 44.2 ± 2.5^#^ |
| **Total cholesterol (mg/dL)** | 68.41 ± 5.31 | 130.34 ± 9.75** | 72.85 ± 7.28 | 246.24 ± 10.54^##^ |
| **Triglycerides (mg/dL)** | 37.45 ± 4.34 | 67.63 ± 6.25** | 39.16 ± 5.25 | 142.18 ± 8.19^##^ |
| **Fasting blood glucose (mg/dL)** | 89.49 ± 8.29 | 96.37 ± 6.46 | 94.38 ± 6.41 | 99.32 ± 8.13 |
| **Fasting blood insulin (ng/mL)** | 1.09 ± 0.32 | 1.31 ± 0.26 | 0.97 ± 0.25 | 2.34 ± 0.36^#^ |
| **IL-6 (ng/mL)** | 395.25 ± 65.3 | 950.54 ± 103.76** | 424.53 ± 126.43 | 2045.75 ± 246.64^##^ |
| **TNF-α (ng/mL)** | 16.32 ± 6.77 | 46.67 ± 8.95* | 18.46 ± 5.03 | 74.66 ± 7.69^#^ |

ND = Normal diet; HFD = High fat diet; ^*^*P*<0.05, ^**^*P*<0.01 versus WT+ND; ^#^*P*<0.05, ^##^*P*<0.01 versus WT+HFD.

All values are presented as mean ± SEM. n = 10-12 in each group.

**Supplementary Figure 1**


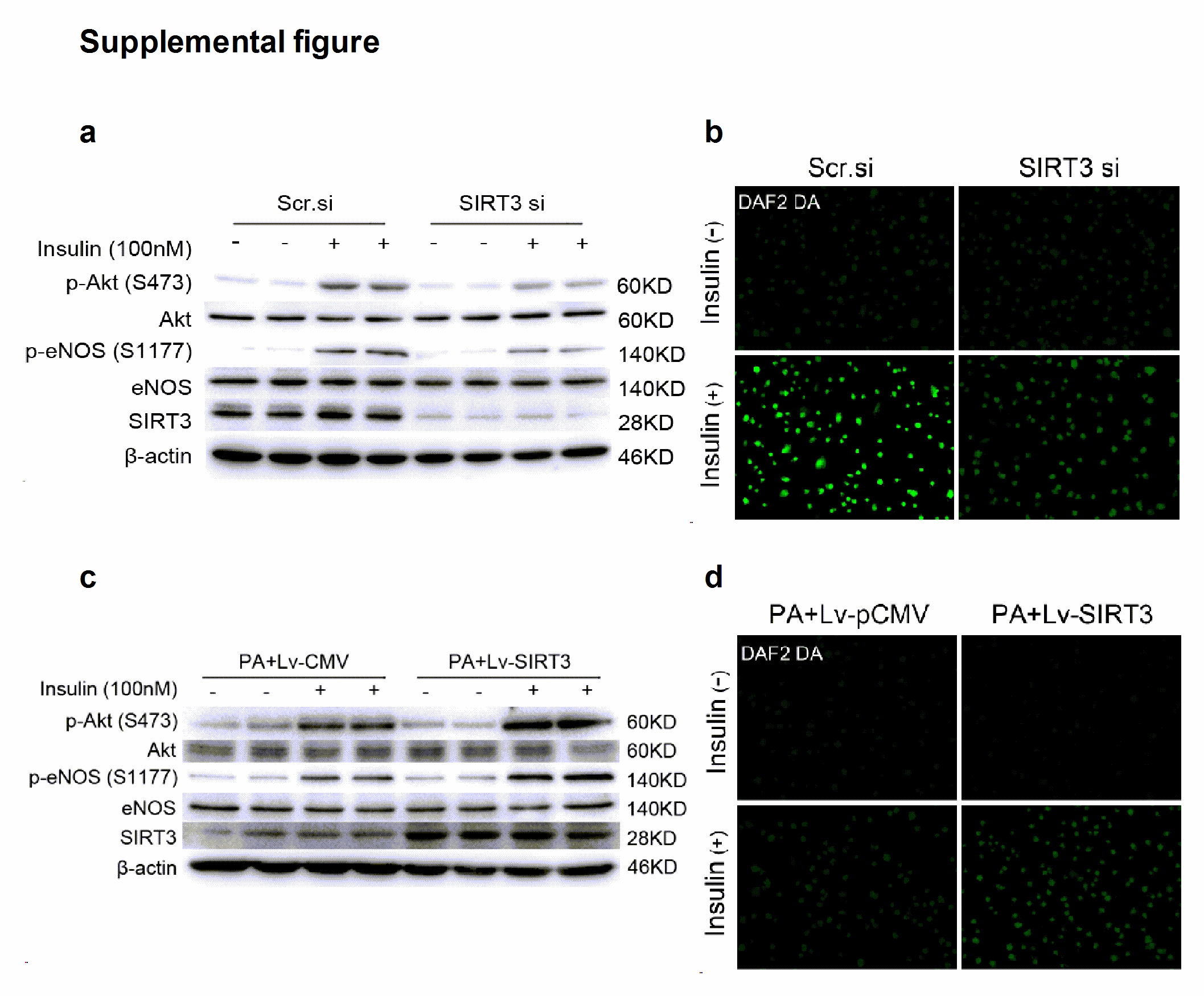


**Figure S1. SIRT3 positively regulated endothelial insulin sensitivity in human aortic endothelial cells (HAECs).** (**a**) The effect of SIRT3 knockdown (SIRT3 si: SIRT3 small interfering RNA; Scr.si RNA: scrambled small interfering) on Akt and eNOS phosphorylation with or without insulin stimulation (100 nM, 20 min) in HAECs was analyzed by Western blot. (**b**) NO production as detected by DAF 2DA fluorescence in HAECs treated with Scr.si or SIRT3 si (magnification, ×100). (**c**) HAECs were infected with lentivirus-SIRT3 (Lv-SIRT3) or negative control lentivirus-pCMV (Lv-pCMV). After 48 h of infection, the cells were exposed to palmitate (PA) for 24 h. The effect of SIRT3 overexpression on Akt and eNOS phosphorylation with or without insulin stimulation (100 nM, 20 min) in HAECs exposed to palmitate (500 nM, 24 h). (**d**) NO production was detected by DAF2DA fluorescence (magnification, ×100) in HAECs with treatment described in C. n = 4 independent experiments.

**Supplementary Figure 2**


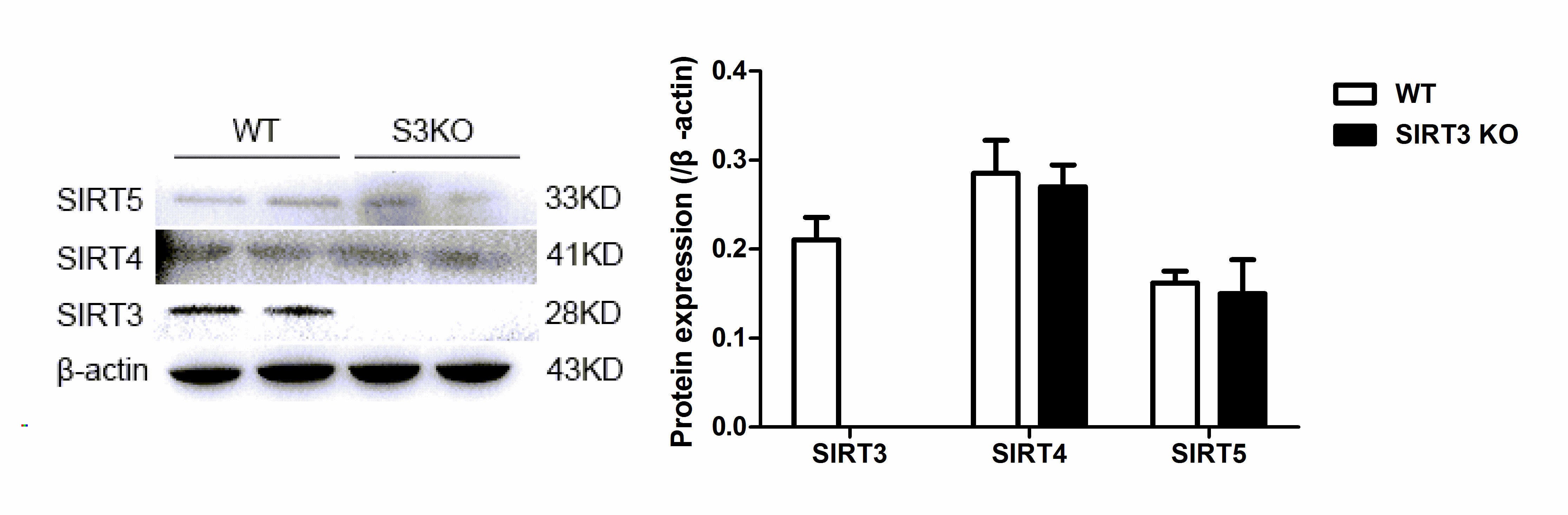


**Figure S2. The effect of SIRT3 deficiency on** **SIRT4 and SIRT5 expression.** Representative Western blot and densitometric quantification of mitochondrial SIRT3, SIRT4 and SIRT5 expression in vessel lysates from WT and SIRT3KO mice. n = 4 independent experiments.

**Supplementary Figure 3**


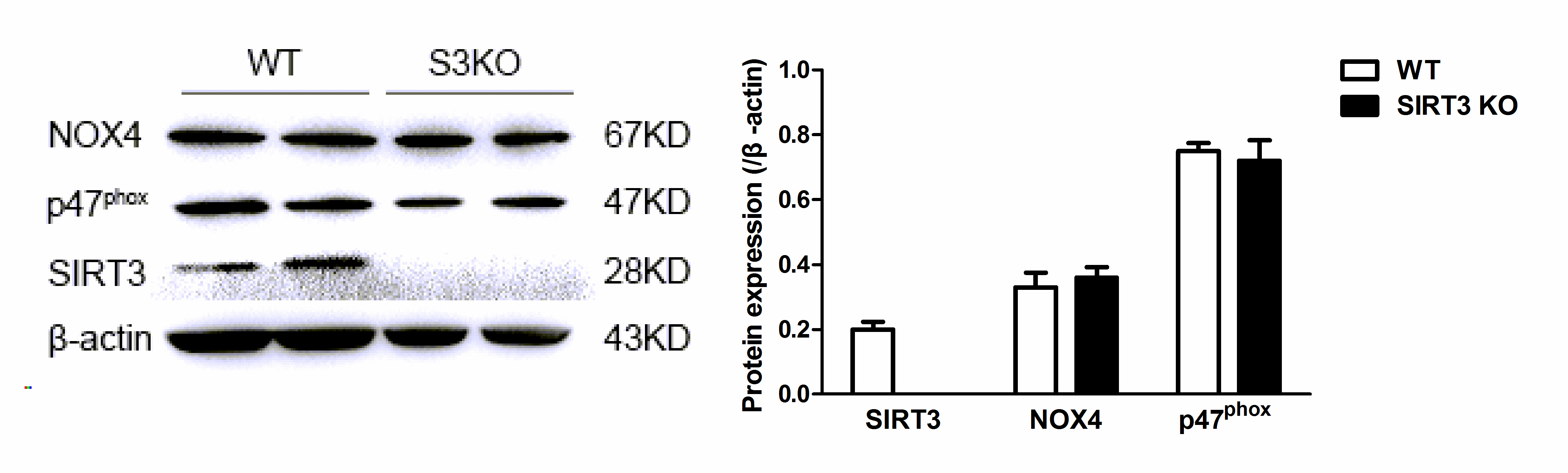


**Figure S3. The effect of SIRT3 deficiency on** **NADPH oxidase 4 (NOX4) and p47phox expression**. Representative Western blot and densitometric quantification of NOX4 and p47phox expression in vessel lysates from WT and SIRT3KO mice. n = 4 independent experiments.
